# DNA methylation analysis: speedup of bisulfite-mediated deamination of cytosine in the genomic sequencing procedure

**Published:** 2004-04-01

**Authors:** Hikoya Hayatsu, Kazuo Negishi, Masahiko Shiraishi

**Affiliations:** *) Shujitsu University, School of Pharmacy, 1-6-1 Nishigawara, Okayama 703-8516, Japan; **) Okayama University Advanced Science Research Center, Department of Genomics and Proteomics, Tsushima, Okayama 700-8530, Japan; ***) National Cancer Center Research Institute, DNA Methylation and Genome Function Project, 1-1, Tsukiji 5-chome, Chuo-ku, Tokyo 104-0045, Japan

**Keywords:** DNA methylation, bisulfite, cytosine deamination, genomic sequencing

## Abstract

Understanding the biological consequences of DNA methylation is a current focus of intensive studies. A standard method for analyzing the methylation at position 5 of cytosines in genomic DNA involves chemical modification of the DNA with bisulfite, followed by PCR amplification and sequencing. Bisulfite deaminates cytosine, but it deaminates 5-methylcytosine only very slowly, thereby allowing determination of the methylated sites. The deamination is usually performed using sodium bisulfite solutions of 3–5 M concentration with an incubation period of 12–16 hr at 50 °C. We demonstrate here that this deamination can be speeded up significantly. We prepared a solution of 10 M bisulfite concentration of pH 5.4 and used it to treat DNA at temperatures up to 90 °C. In an experiment, in which denatured DNA was treated with 9 M bisulfite for 10 min at 90 °C, deamination of cytosines occurred to an extent of 99.6%, while 5-methylcytosine residues in the DNA were deaminated at less than 10%. Using a plasmid DNA fragment, we observed that the DNA can serve as a template for PCR amplification after the bisulfite treatment. This new procedure is expected to offer a significantly improved genomic sequencing method, leading to the promotion of research on understanding the biological and medical significance of DNA methylation.

## Introduction

Bisulfite-mediated deamination of cytosine is a chemical reaction discovered in 1970.1)–3) It serves as a unique method to cause hydrolytic deamination selectively in the cytosine moiety among nucleic acid bases, and has been used extensively in studies of DNA and RNA (see ref. 4) for review). Since methyl-substitution at position 5 of cytosine makes the amino group at position 4 resistant to the bisulfite-deamination,3) this reaction has been used for discriminating 5-methylcytosine residues in DNA from those of cytosine.5)

In studies on the functional consequences of methylation in DNA, ‘bisulfite genomic sequencing’ is a principal methodology for determining the methylated sites.6) A recommended procedure involves a treatment of DNA with 4.9 M sodium bisulfite at pH 5 and at 50 °C for 12–16 hr.7) The subject of DNA methylation is of great current interest in biology and in medical sciences.7),8) It is anticipated that the DNA methylation may be used as a biomarker for clinical diagnosis of cancer8); for example, use in early detection and in monitoring therapeutic efficacy. We have now investigated this chemical method with the purpose of shortening the time of bisulfite treatment. Shorter periods of treatment would be desirable to minimize the oxidative decay of bisulfite, which can result in acidification of the reaction mixture, causing depurination and chain breakage of DNA.

**Fig. 1:**
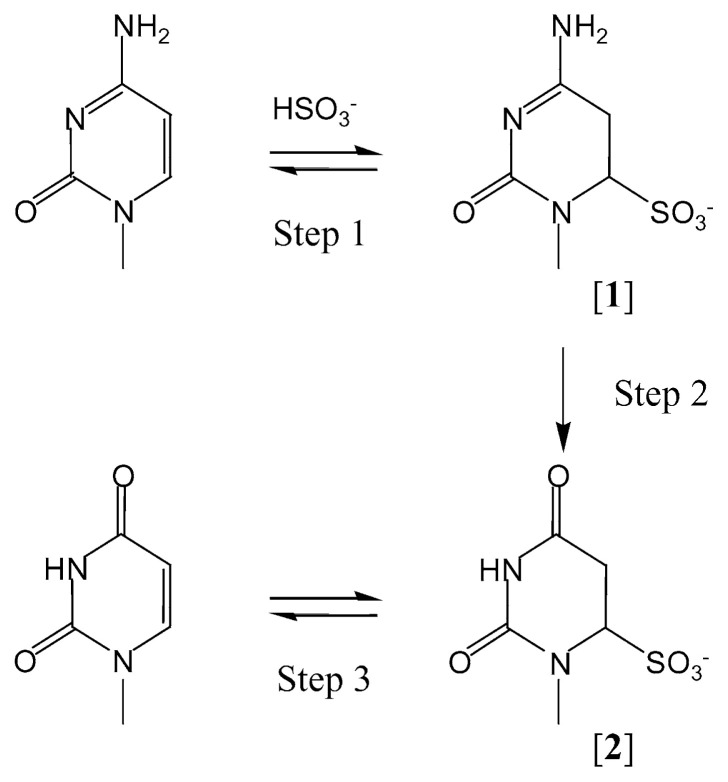
Bisulfite-mediated deamination of cytosine.

Cytosine, either as a base, a nucleoside, or a constituent of polynucleotides, can be converted into its 5,6-dihydro-type adduct [1] when mixed with bisulfite in aqueous solutions (step-1 in Fig. 1).4) Step-1 is an equilibrium that can be attained rapidly. In solutions of 1 M sodium bisulfite, for example, adduct-1 is predominant at acidic pHs (pH 4–6) but not at pHs higher than 7.3) Adduct-1 then undergoes hydrolytic deamination to give the uracil bisulfite adduct [2] (step-2), which can be converted to uracil on treatment with mild alkali (step-3). The optimum pH for the overall conversion, cytosine to adduct-2, is around 5. The rate-determining step for the overall reaction is step-2. Previous studies on factors governing the rate of deamination have revealed that the rate is approximately proportional to the bisulfite concentration, 9) suggesting that sulfite participates in step-2 in addition to step-1.9),10) Therefore, an increase in the bisulfite concentration of the reaction solution could result in a significant acceleration of the deamination. Higher temperatures are also expected to be favorable for more rapid deamination, although lower temperatures are more favorable for adduct-1 formation in step-1.3) Here, we wish to report efficient deamination of 2′-deoxycytidine by use of solutions of high bisulfite concentrations up to 10 M. Use of the method for deaminating cytosine in DNA is also described.

## Materials and methods

### Materials

Sodium bisulfite, sodium sulfite (Reagent Grade), ammonium sulfite monohydrate, 50% ammonium bisulfite solution, and 0.1 N HCl were purchased from Wako (Osaka, Japan). Nucleosides and salmon testis DNA were products of Sigma (St. Louis, MO, USA). Plasmid pUC119 was obtained from TaKaRa (Kyoto, Japan). Restriction endonuclease *Sca*I was obtained from New England BioLabs (Beverly, MA, USA).

### General methods

Reactions and manipulations at desired temperatures were performed using, unless otherwise noted, thermostatted water baths with an accuracy of ± 0.1 °C (except for reactions at 90 °C, in which the temperature was maintained at ± 1 °C). Solubilization of reagents in aqueous media was done by warming the mixture at a desired temperature for 5–10 min with frequent vigorous shaking. For spectrophotometry, a Hitachi model U-2800 UV-Vis spectrophotometer (Tokyo, Japan) was used.

### Determination of bisulfite concentrations

To assay concentrations of bisulfite in solutions, we devised a spectrophotometric method based on the absorbance of sulfur dioxide in acidic solutions, i.e., absorbance at 276 nm in 0.1 N HCl (molar extinction coefficient, 380 ± 20).11) Thus, a bisulfite solution was appropriately diluted with water, and 30 μl of the diluted solution was added to 3.0 ml 0.1 N HCl that had been placed in a cuvette (1.0 × 1.0 × 4.4 cm). The cuvette was then covered with Parafilm and hand-shaken up-sidedown 3 times for mixing, and the A_276_ was measured. 1 M Na_2_SO_3_ was used as a standard: the A_276_ for this standard showed a linear dose-response at 0.5 mM-3 mM (A_276_ 0.2–1.2).

### Reactions of cytosine nucleosides with bisulfite

For reaction rate measurements, the deamination extents were determined spectrophotometrically as described previously.9) Briefly, one volume of 0.20 M 2′-deoxycytidine was mixed with 10 volumes of a bisulfite reagent; usually, a 275 μl reaction mixture was prepared and placed in a tightly stoppered 1.5 ml Eppendorf tube. Individual tubes, each of which was assigned to a desired period of reaction time, were prepared. The reactions were terminated by addition of 500 μl cold water. A sample (75 μl) of the diluted solution was taken and added to 5 ml 0.2 M sodium phosphate buffer of pH 7.2. After allowing the solution to stand at room temperature for 40 min, A_270_ was determined. Decrease in A_270_ from an appropriately prepared untreated-sample (A_270_, about 0.8) is the measure of deamination (100% deamination results in an A_270_ value less than 0.05, with a sulfite-only control being taken into account (~0.05 A_270_ for 9 M bisulfite)). With 5-methyl-2′-deoxycytidine, A_277_ was employed in place of A_270_ for the rate measurement.

To determine the pH dependence of the rate, bisulfite solutions of various pH values were prepared by dissolving mixtures of NaHSO_3_ and Na_2_SO_3_ (10 mmoles in total, at different ratios) into 50% ammonium bisulfite solution to make a final volume of 10 ml. The bisulfite content found for the prepared solutions was 7 M.

### HPLC analysis of nucleosides

The analysis was performed using a Hitachi L-7100 pump (Tokyo, Japan), a Beckman-Coulter column Ultrasphere ODS 4.6 mm × 25 cm (Fullerton, CA, USA), and a 260 nm UV-detector. Ten μl of sample was injected, and eluted with a linear gradient composed of buffers A and B (100% A at 0 time, 100% A at 5 min, 85% A at 20 min, 55% at 35 min, and 0% at 60 min) at a flow rate of 0.7 ml/min using 100 mM K-phosphate, pH 7, as buffer A, and 90% methanol-1 mM K-phosphate, pH 7, as buffer B. The retention time of standard nucleosides in this HPLC was 19 min for 2′-deoxycytidine, 22 min for 2′-deoxyuridine, 25 min for 5-methyl-2′-deoxycytidine, 26 min for 2′-deoxyguanosine, 28 min for thymidine, and 32 min for 2′-deoxyadenosine.

### Bisulfite treatment of DNA

#### (1) Treatment and analysis of base composition

Salmon testis DNA denatured in 0.3 N NaOH for 30 min at 30 °C was mixed with 10 volumes of 10 M (NH_4_)HSO_3_-NaHSO_3_ solution, pH 5.4. The mixture was incubated using a TaKaRa PCR thermal cycler, first at 95 °C for 30 sec and then at a desired temperature. Upon completion of the treatment, the sample containing 80 μg of DNA in 605 μl in volume was applied onto Sephadex G-50 column (**φ**15 × 40 mm, Bio-Rad Econo-Pak10) equilibrated with 10 mM Tris-HCl-1 mM EDTA, pH 8, and eluted with the same buffer to obtain a DNA fraction. DNA was collected by precipitation with ethanol and was dissolved in 100 μl of H_2_O. To generate uracil from uracil bisulfite adducts, 90 μl of the sample solution was added to 11 μl of 2 N NaOH, and incubated for 10 min. DNA was collected by ethanol precipitation, digested with DNase I (Boeringer), snake venom phosphodiesterase (Worthington) and calf intestinal alkaline phosphatase (Promega) into nucleosides, and submitted to HPLC analysis.

#### (2) Bisulfite treatment and amplification of treated DNA by polymerase chain reaction (PCR)

One μg of *Sca*I-digested pUC119 DNA was treated with 50 μl of 0.3 N NaOH for 30 min at 37 °C. The reaction tube was then placed in a water bath maintained at either 70 °C or 90 °C for 3 min. Five hundred μl of 10 M bisulfite, pH 5.4, maintained at the same temperature was added to the DNA solution, mixed with pipetting, and mineral oil was overlaid. At a desired time of incubation, a sample of 130 μl was removed, mixed with water, and kept on ice. DNA was recovered using Wizard DNA Clean-UP System (Promega, Madison, WI, USA) following the manufacturer’s recommendation and finally dissolved in 90 μl of water. Eleven μl of 2 N NaOH was added to the DNA solution and the resulting mixture was incubated at 37 °C for 10 min. DNA was recovered by ethanol precipitation, with yeast tRNA (10 μg, Sigma) added as a carrier, and was dissolved in 100 μl of TE (10 mM Tris-HCl, pH 7.5, plus 1 mM EDTA, pH 8.0).

One μl of this DNA solution was subjected to the PCR reaction. AmpliTaq DNA polymerase Stoffel fragment (Applied Biosystems, Foster City, CA, USA) was used following the instructions of the manufacturer. After a 3-min incubation at 95 °C, 30 cycles of amplification (30 sec at 95 °C; 30 sec at 57 °C; 3 min at 70 °C) was performed. The PCR primers used were 5′-CGGAATTCTATTGGTTAAAAAATGAG-3′ and 5′-AACTGCAGACATTAACCTATAAAAATA-3′. The reaction volume was 50 μl. One μl of this PCR product was subjected to agarose gel electrophoresis.

## Results and discussion

### Bisulfite contents in reagents

By using Na_2_SO_3_ as a standard for determining bisulfite content of a given reagent, we observed that the NaHSO_3_ (an agent with a remark on its label stating it is a mixture of sodium bisulfite, NaHSO_3_, and sodium metabisulfite, Na_2_S_2_O_5_) contained 1.11 mol of bisulfite per mol formula-weight (namely, the sample was mostly Na_2_S_2_O_5_), and that the (NH_4_)_2_SO_3_≥H_2_O contained 0.92 mol bisulfite per mol formula-weight. 50% Ammonium bisulfite solutions (3 different bottles tested) were found to contain 6.0–6.2 M bisulfite.

### Preparation of bisulfite solutions

First, we determined the highest bisulfite concentrations obtainable by dissolving sodium bisulfite, sodium sulfite or ammonium sulfite in water. Thus, NaHSO_3_ can be dissolved up to 5.9 M at 70 °C, and 5.0 M at 30 °C; Na_2_SO_3_ to 2.1 M at 70 °C, and 1.5 M at 30 °C; and (NH_4_)_2_SO_3_≥H_2_O to 4.3 M at 70 °C and 3.5 M at 30 °C. The highest bisulfite concentration obtainable by dissolving a mixture of NaHSO_3_ and Na_2_SO_3_ at 70 °C to give a solution of pH 5.4 was 5.9 M.

Next, the availability of 50% ammonium bisulfite solution from a commercial source enabled us to prepare solutions of higher bisulfite concentrations. By dissolving a mixture of 2.08 g NaHSO_3_ and 0.67 g (NH_4_)_2_SO_3_≥H_2_O in 5.0 ml 50% (NH_4_)HSO_3_ (pH 4.5) by heating at 70 °C for 5–10 min, a solution of pH 5.4 containing 10 M bisulfite was obtained (final volume, about 6 ml). This solution is stable: thus, when a freshly prepared sample was allowed to stand in a tightly stoppered container at 70 °C, the bisulfite content and the pH value showed no change after 4 hr.

### Reactions of 2′-deoxycytidine with bisulfite

**Fig. 2:**
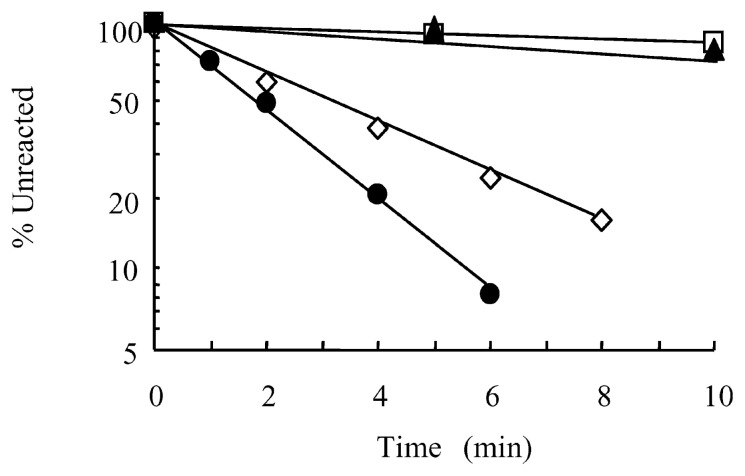
Deamination of 2′-deoxycytidine at 70 °C and pH 5.4: ● 9 M (NH_4_)HSO_3_-NaHSO_3_, ⋄ 5.3 M NaHSO_3_-Na_2_SO_3_. Data for 5-methyl-2′-deoxycitidine are also shown: 9 M (NH_4_)HSO_3_-NaHSO_3_ at 90 °C (▲) and at 70 °C (□).

Fig. 2 shows typical time courses of deamination occurring in bisulfite reagents at pH 5.4. The reactions proceeded with pseudo-first-order kinetics. The half life (t_1/2_) of deoxycytidine at 70 °C in 5.3 M NaHSO_3_-Na_2_SO_3_ was 3.0 min, while that in 9 M (NH_4_)HSO_3_-NaHSO_3_ was 1.8 min. Thus, the rate of deamination (1/t_1/2_) was indeed proportional to the bisulfite concentration: [t_1/2_ at 5.3 M]/[t_1/2_ at 9 M], 1.7, was equal to [9 M]/[5.3 M]. This proportionality was also observed for reactions conducted in bisulfite solutions stepwise-diluted from 9 M bisulfite (data not shown). The half lives of deoxycytidine in 9 M bisulfite, pH 5.4, at temperatures other than 70 °C were as follows (half life/temperature): less than 1 min/90 °C, 5 min/50 °C, and 17 min/37 °C. Deamination of 5-methyl-2′-deoxycytidine in 9 M (NH_4_)HSO_3_-NaHSO_3_ was slow both at 70 °C (16% at 10 min) and at 90 °C (23% at 10 min)(Fig. 2).

Quantitative conversion of 2′-deoxycytidine into 2′-deoxyuridine in these reactions was confirmed by HPLC analysis. Thus, after the bisulfite treatment, the mixture was treated with alkali, and subjected to HPLC analysis. We observed 100% conversion of 2′-deoxycytidine into 2′-deoxyuridine for reactions in 9 M bisulfite at pH 5.4, both in a reaction at 90 °C for 8 min and a reaction at 70 °C for 30 min.

**Fig. 3:**
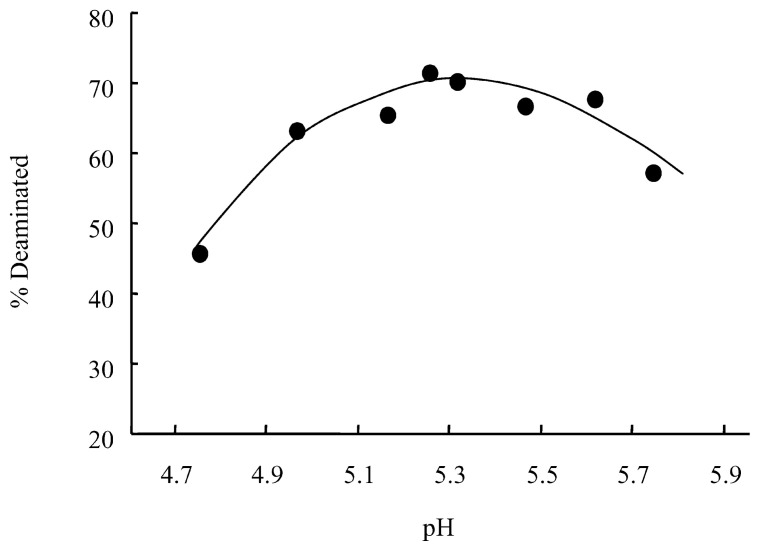
pH-rate profile for deamination of 2′-deoxycytidine in 7 M bisulfite. Reaction extents at 60 °C and 5 min are plotted.

The pH dependence of the deamination was determined using 50% ammonium bisulfite containing 1 M sodium bisulfite at pH 4–6. The temperature employed in this experiment was 60 °C, at which the reactions are slower than at 70 °C to allow more accurate determination of the reaction extents. As Fig. 3 shows, the optimum pH-range is 5.0–5.6, consistent with that previously reported for the deamination of cytidine 5′-phosphate in 2 M sodium bisulfite at 37 °C.9) The wide range of optimum pH is obviously advantageous for avoiding slowdown of the reaction during incubation.

Hydroquinone, which has been used previously for the prevention of bisulfite decay,7) is unnecessary in the present experiments that require only much shorter periods of incubation than those conventionally employed.

### Reactions of DNA with bisulfite

**Fig. 4:**
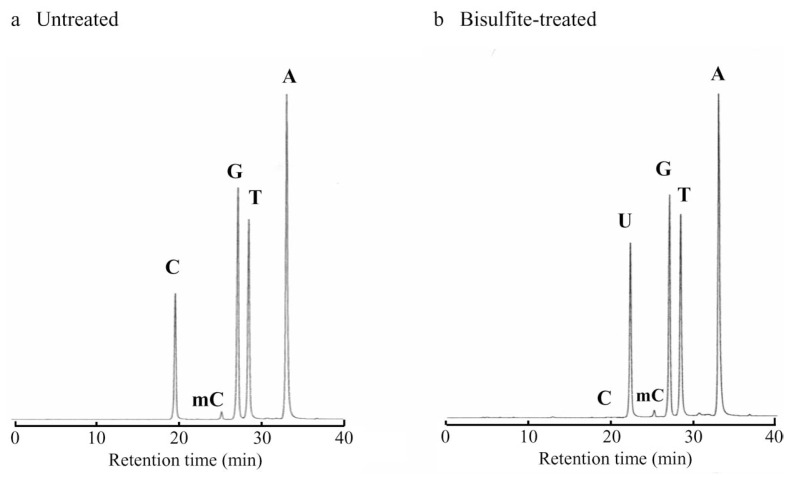
Analysis of DNA nucleosides by HPLC. *a* Untreated DNA; *b* DNA treated with 9 M bisulfite at 90 °C for 10 min: C, 2′-deoxycytidine; U, 2′-deoxyuridine; mC, 5-methyl-2′-deoxycytidine; G, 2′-deoxyguanosine; T, thymidine; A, 2′-deoxyadenosine.

Salmon testis DNA was treated with 9 M bisulfite. As shown in Fig. 4 and Table I, a treatment at 90 °C for 10 min resulted in a virtually complete deamination of cytosine in the DNA (99.6% of C), while 5-methylcytosine was only little affected (less than 10% deamination) and the other bases were unaffected (a small decrease in the guanine content may require further study to determine its significance). Almost complete deamination of cytosine was also observed in the treatment of the DNA at 70 °C for 16 min or at 37 °C for 170 min.

For this efficient deamination to be useful in genomic DNA sequencing, it is required that the bisulfite-treated DNA can be amplified by PCR. We treated a cloned DNA fragment with the 9 M bisulfite at 90 °C for 10 min, the DNA was recovered, and then investigated whether the treated DNA fragment can serve as a template for PCR amplification. Amplified fragments were detected in approximately equal amounts for DNA samples that had undergone treatments at 90 °C or at 70 °C for periods of 5, 10, 20 and 40 min (data not shown). This observation indicates that the DNA did not undergo serious degradation during these treatments.

We expect that this new deamination procedure would be useful in speeding up the analysis of methylated sites in DNA. Such a significant improvement in the bisulfite genomic sequencing will greatly promote research on the biological and medical consequences of DNA methylation.

## Figures and Tables

**Table I:** Base composition of salmon testis DNA treated with 9 M bisulfite at 90°C for 10 min (as determined by HPLC shown in Fig. 4)

	Mol %					

	C	U	mC	G	T	A
Untreated	20.26	0.04	1.41	22.43	28.67	27.19
Bisulfite-treated	0.08	19.89	1.29	21.56	29.28	27.90
